# Valve-like dynamics of gas flow through a packed crystal mush and cyclic strombolian explosions

**DOI:** 10.1038/s41598-018-37013-8

**Published:** 2019-01-29

**Authors:** Anna Barth, Marie Edmonds, Andrew Woods

**Affiliations:** 1 Department of Earth Sciences, University of Cambridge, Downing St, Cambridge, CB2 3EQ United Kingdom; 2 BP Institute, University of Cambridge, Madingley Rd, Cambridge, CB3 0EZ United Kingdom

## Abstract

Strombolian volcanic explosions are commonly attributed to the rise and burst of conduit-filling gas slugs. The magmas associated with strombolian activity, however, are typically not only volatile-rich but also highly crystalline, with mush regions in the shallow plumbing system, where an exsolved volatile phase may also be abundant. Through analogue experiments, we explore a new mechanism to form gas slugs and strombolian explosions. A steady flux of gas is supplied to the base of a particle-rich liquid layer, generating a localised gas intrusion, which initially grows through plastic deformation. Once the pressure in the intrusion overcomes the effective tensile strength of the particle pack, a localised channel opens, allowing gas to propagate upwards. As the pressure in the intrusion falls, the gas pocket collapses. The continued supply of gas leads to the formation of a new intrusion, and the cycle repeats. With higher gas fluxes, continuous channelised gas flow occurs. Highly crystalline shallow portions of basaltic conduits may act as a flow valve, transforming a steady gas flux into a series of discrete gas slugs which cause explosions.

## Introduction

### A link between strombolian activity and crystal-rich magmas

The periodicity of typical strombolian eruptions has been attributed to the rise and bursting of large, conduit-filling, over-pressurized gas slugs^1–4^. At Stromboli Volcano, Italy, the type example of such activity, the gas slugs are often rich in CO_2_ over more soluble volcanic gases^5,6^, indicating an origin of a few kilometres depth or more^7^, where extensive crystal-rich magma reservoirs are inferred to exist^8–10^. Magmas erupted from Stromboli Volcano are highly crystalline, containing up to 40 vol% phenocrysts^11–13^ and it is likely that the exsolved gas phase interacts with the crystal cargo of the magma^14–16^, thereby modifying the dynamics of the eruptions. Other volcanoes which exhibit “classic” strombolian behaviour are Yasur Volcano (Vanuatu)^17^, which erupts magmas containing 35–40 vol% crystals^10^; Erebus Volcano (Antarctica)^18^, where magmas contain 30 vol% anorthoclase feldspar^19^; and other crystal-rich basaltic-andesitic arc centres, such as Pacaya^20^, Fuego^21^, Pavlov^22^, and Karymsky^23^.

Volcanoes which exhibit classic strombolian eruptive behaviour are often in subduction zone or continental rift settings, where melts contain substantial (>1 wt% and often up to 3–4 wt%) water concentrations, giving rise to extensive degassing-induced crystallization^24^ and consequent rheological changes in the magmas as they ascend the last km or so toward the surface. The rapid increase in viscosity due to water exsolution and crystallization may give rise to a degassed plug, where gas may accumulate before exceeding the tensile strength of the plug^16^. The magmas that are involved in strombolian eruptions are extremely volatile-rich, and give rise to these eruptions that may be thought of as essentially gas eruptions, with very little magma. Owing to the high carbon dioxide content of such magmas (up to 2 wt%^6^), they may become vapor-saturated at pressures of 300 MPa or higher in the crust, allowing a substantial exsolved volatile phase to form at upper crustal depths of a few kilometers. At Stromboli volcano, for example, the CO_2_-rich gas that plays such an important role in the formation of the gas slugs which drive strombolian explosions is thought to be derived from >4 km depth (Burton *et al*., 2007) and the larger, less frequent paroxysms may be driven by hot, CO_2_-rich magma at depths of a few kilometers which may stall in sills, allowing efficient gas segregation and rise, perhaps triggering explosive activity^6,25^.

The relatively small volume of magma erupted during typical strombolian eruptions, combined with the high mass flux of emitted volcanic gases, requires that degassed, highly crystalline magmas are stored in the plumbing systems of such volcanoes. At Yasur volcano, Vanuatu, for example, it has been estimated that ~25 km^3^ of degassed basaltic-trachyandesitic magma (containing ~1 wt% H_2_O) must have ascended to at least 1 km depth, degassed, then was stored as a shallow cumulate body beneath the scoria cone, over the past 1000 years^26^, contributing to block resurgence in the Siwi Caldera. At Stromboli Volcano, the mean, time-averaged, magma supply rate to shallow depths in the conduit, in order to supply the observed outgassing rates of sulfur and chlorine, is ~0.3 m/s^27^; and 15 times more magma is degassed and stored, than is erupted. This volume of stored, highly crystalline magma amounts to 0.25 km^3^ over 30 years^27,28^. These gas-rich strombolian eruptions that typify basaltic to andesitic magmatic systems in arc settings may therefore be thought of as the surface manifestations of large, intrusive, crystal-rich magma bodies emplaced in the shallow crust.

At Stromboli, the prevailing picture from petrological studies^8^ suggests that crystal-rich mush exists throughout the depth interval 2–4 km^8^. Ca-basaltic melts entrapped in high-Mg olivines (Fo_89–90_) generate the erupted basalts through crystal fractionation. The Stromboli plumbing system is probably a succession of magma ponding zones connected by dykes. The 7–10 km interval, where primitive magmas are stored and differentiate, is periodically recharged by new magma batches, possibly ranging from Ca-basalts to basalts, with an exsolved CO_2_-rich gas phase^6^. These deep recharges promote the formation of bubbly basalt blobs, which are able to intrude the shallow plumbing system (2–4 km), where CO_2_ gas fluxing enhances H_2_O loss, crystallization and generation of crystal-rich, dense, degassed magma. This cumulate body, or crystal mush, has built up beneath Stromboli over the past 2.0–2.5 ka^13,29^. The existence of this crystal-rich body is evidenced by the presence of inherited crystals (antecrysts), recording complex zoning and dissolution surfaces related to repeated interactions with the ascending LP magma, which are systematically found in pumices^9,29^. This cumulate body or mush, to which is contributed the recycled, degassed HP basalt, possibly acts as a filter press, allowing the low-porphyricity, CO_2_-rich melts that trigger the paroxysms to rise up through it, or interact with it. It is likely then that exsolved CO_2_-rich gases must travel through this crystal-rich magma storage region, in order to reach the shallow conduit and surface.

Up to now, the formation and rise of an exsolved volatile phase in the form of bubbles and slugs to form strombolian activity has been almost exclusively treated as a two phase system (melt and bubbles)^2,30–32^ (recent exceptions are described below). Given that up to 35–40 vol% macrocrysts (large phenocrysts) exists in erupted magmas from many of these volcanoes, as well as the extensive petrological evidence (described above) for crystal reworking from mush-like regions in the edifice and deeper^8^, is this simple treatment realistic? How important might the crystal phase be both in controlling the dynamics and behaviour of gas slugs in the conduit but also much deeper in the system, for generating periodic release of an exsolved volatile phase at depth? The interaction between an exsolved volatile phase and crystal-rich magmas at depth might even be a key mechanism to allow the exsolved volatile phase to segregate from magma bodies to allow it to migrate to the surface.

There have been numerous recent studies that focus on these questions, using both analogue and numerical modeling, which have produced our first hints of understanding the interactions between gas and particle suspensions. The behaviour of bubbles of gas upon encountering particles suspended in a viscous liquid was studied by^14^. They showed that large bubbles break up and rise around particles as smaller bubbles; but smaller bubbles may become trapped in the particle pack. The relative size of particles and crystals may be an important control on their behaviour and whether gas may be ‘held up’ within a crystal-rich layer^14^. It has been observed, in number of studies, that when gas is supplied to a particle-water system in a Hele-Shaw cell, the gas may open channels through the pack^33,34^. This work was extended by^15^, who conducted analogue experiments to investigate gas migration regimes within particle-rich suspensions, while varying both particle fractions and liquid viscosities. It was found that the particle packing fraction was the critical parameter in determining gas migration regime. At low particle fractions, the bubbles deform around the particles, producing slow migration of gas where gas flows around individual particles; at high particle fractions, the mixture tends to fracture in a quasi-brittle fashion^15^, allowing gas to flow rapidly through channels. It was speculated that such behaviour could lead to repeated bursts of degassing^15^.

Analogue experiments in the clay-water system (aimed at understanding geological applications of clay seals) have also shown similar fracture-like behaviour^35^, but also highlighted the development of cyclicity in gas transport associated with the continuous supply of gas to the system. Air was injected into the center of a thin cylindrical cell initially filled with a mixture of bentonite clay and water^35^. For relatively dry mixtures (the behaviour was not observed above a particular water content of the clay-water mixture), the pressure initially increases with little volume change, but on reaching the yield stress of the clay-water mixture, the lid of the cell then deforms elastically and an air-filled void forms in the center of the cell as the clay is driven radially outward. With continued supply of air, the pressure continued to increase until reaching the fracture strength of the clay, whereupon a fracture-like channel formed and migrated to the outer edge of the cell, enabling the air to escape. The pressure then falls, and the clay flows back toward the center of the cell and seals the channel so the cycle can repeat.

Numerical modeling has provided insights into how an exsolved volatile phase may travel through a crystal-rich mush. The idea that an exsolved volatile phase may migrate into crystal-rich magmas from underplating basaltic magmas was proposed by^36^ and later expounded upon by^37,38^. In a crystal-rich magmatic environment, it is proposed that that the exsolved volatile phase exhibits a viscous fingering behaviour, allowing it to migrate relatively rapidly through crystal-rich mush. It is argued that the exsolved volatile phase, during transport, does not deform the mush at pressures of greater than 1.5–2 kbar; deformation of the particle pack at high confining pressures may require unrealistically high shear stresses^38^, although capillary fracturing and veining may occur at higher particle fractions, to allow the extensive gas loss from plutonic bodies of magma^39^. Models of gas overpressure beneath a degassed, crystal-rich plug shows that cyclic strombolian eruptions may be generated at Stromboli by the failure and fracturing of the plug as the overpressure in a trapped gas pocket increases^16^. The plug may exhibit both ‘flow’ and ‘fracture’ regimes depending on the supply and accumulation of gas bneath it, thereby accounting for both passive degassing and explosive behaviour^16^.

The mechanisms by which an exsolved volatile phase may interact with and migrate through such a crystal-rich region of the magmatic system, however, remain unclear. There is potential for this interaction between crystal-rich magma and gas to modulate gas release into the shallow parts of the eruptive system and to play a large role in controlling strombolian eruption dynamics that has, up to now, has been underappreciated. In this paper, we explore the buoyancy-driven flow of gas through a packed bed of particles in a Hele-Shaw cell, as a model for the gas flux through a crystal-rich layer in a magmatic system, which may be a mush^40^ in a region of a shallow magma reservoir. In particular, we explore experimentally whether gas slugs may be generated through the interaction of an exsolved volatile phase with a highly crystalline layer; the range of regimes and phenomena that may be produced under conditions of variable gas flux; and whether this mechanism may provide a generic model for cyclic gas release and hence strombolian eruptions.

### Analogue experiments to elucidate gas transport through a crystal-rich layer

**Figure 1:**
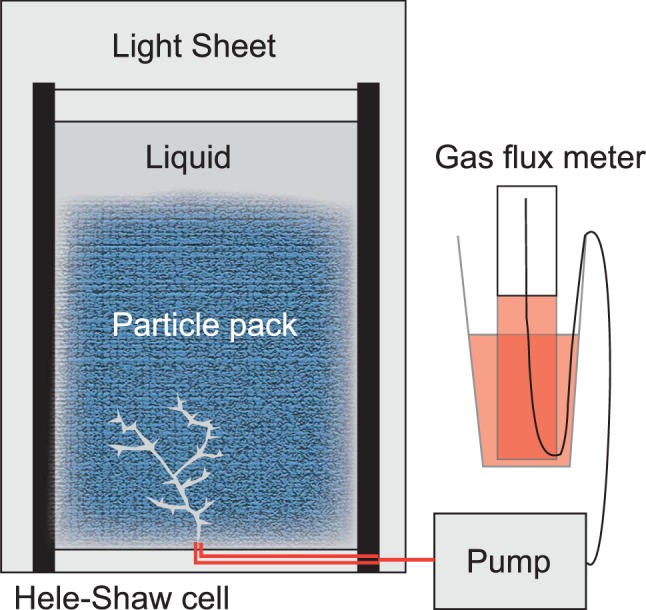
The experimental set-up to explore interaction of gas with a particle pack, an analogue for a crystal-rich mush. A peristaltic pump was used to inject air, at variable rates, into the base of a Hele-Shaw cell filled with liquids of varying viscosity. The cell was packed, 40 cm high, with spherical cellulose acetate particles of density 1290 kgm^−3^.

A particle pack of depth 40 cm was set up between two parallel sheets of Perspex, 1 cm apart, and filled with a solution of Natrasol polymer and water, of density 1000 kg/m^3^ (Fig. 1). Four different solutions were used, with viscosities ranging from 0.22, to >5 Pa s. The particles (plastic spheres of radius 1 mm) have density 1290 kg/m^3^ which leads to a Stokes settling speed of order 10^−5^ m/s in the solution of Natrasol, and the packing fraction is 60–65 vol%. Air was supplied at the base of the particle pile using a calibrated peristaltic pump to supply a known range of gas fluxes, at injection rates of up to 2 mL/s. Flow rate of the gas was monitored using high temporal resolution images of the liquid height of a closed cylinder of liquid as air was pumped from the headspace. High-resolution digital photographs recorded the motion and deformation of the particle pile in response to the gas flux. Time lapse imagery was acquired and analysed to monitor the evolution and form of the channels and fractures that developed in the particle pack. See methods for a detailed description of the experimental set-up and analysis.

The key observation from the experiments is that gas injection at a constant rate at the base of a granular suspension gives rise to periodic release of the gas at the top surface. The gas pocket rises through a series of opening and closing channels, stalling intermittently beneath strong particle structures. Once the gas is released at the top of the pack, the pile collapses back under the force of gravity. Different behaviours are observed according to gas injection rate.

**Figure 2:**
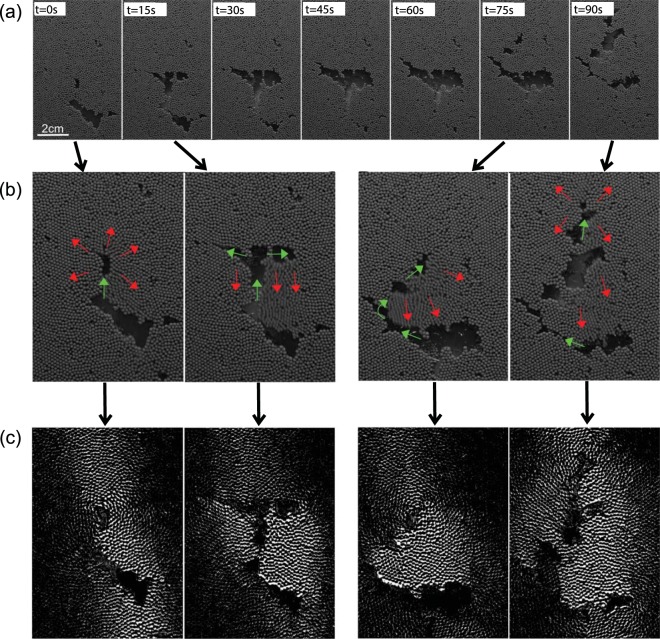
Growth and migration of gas pockets in the particle pack at low gas fluxes. (**a**) Series of photographs to illustrate the successive growth and migration of three gas pockets within the bead pack as a result of the continuing gas flux. The time between frames in this image is 15 seconds and the gas flux was 0.1 ml/s. The pocket ascends through the pile, gradually dissipating its overpressure until reaching a point where it stalls (at t = 15–60 s). At ~75 s the pocket has grown and become sufficiently overpressured that it can overcome the tensile stress of the particle pack and the gas continues its ascent. (**b**) Enlarged images to illustrate deformation of the particle pack, and flow of gas. Green arrows indicate gas motion; red arrows indicate particle movement. (**c**) Time lapse images show the particles which have moved (bright) and particles which are nearly stationary (dark) corresponding to the images shown in panel b.

**Figure 3:**
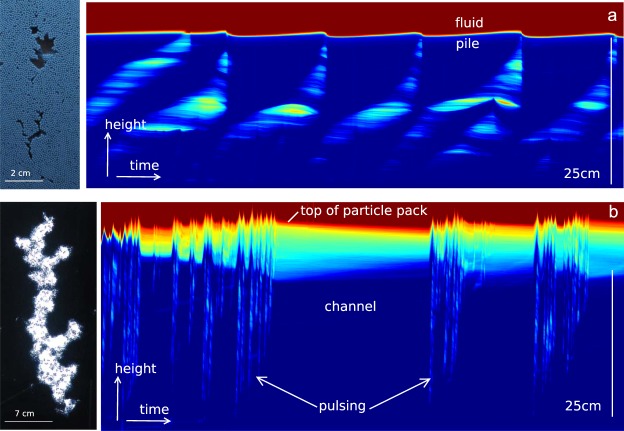
A vertical slice through the particle pack plotted with time, to show the temporal evolution of gas pockets and channels. (**a**,**b**) Evolution of the horizontal average of the light intensity in the bead pack, as a function of vertical position (y-axis) and time (x-axis). The images are shown in false colour with blue indicating beads, and green indicating presence of a bubble. In (**a**), the gas flux is 0.1 cc/s while in (**b**) the gas flux is 1 cc/s. The entire experiment (x axis) lasts for one hour.

Figure 2 illustrates the results of the experiments for a low input gas flux. Gas accumulates near the base of the particle pile, forming an intrusion, or gas pocket. As the gas intrusion grows, the surrounding particle pack deforms plastically on a length scale that is larger than the individual particles (Fig. 2b,c). Eventually, the pressure in the gas intrusion exceeds the tensile strength of the surrounding particle pack and a narrow fracture-like channel opens up, along which the gas can migrate, draining the intrusion, which then closes up (Fig. 2a). The advancing gas may form a new intrusion higher in the particle layer. Meanwhile, the continuing supply of gas at the base then initiates a new intrusion deeper in the cell. The successive formation of gas pockets can be illustrated by plotting the average light intensity with height in the cell (y-axis) against time (x-axis). We show two time series in Fig. 3 for the case of (a) a low gas flux, with intermittent intrusion formation and migration and (b) a higher gas flux, with channelised gas flow. Figure 3a illustrates the formation of gas pockets in the particle pack, which eventually drain, with more gas moving upward to replenish them, or form new pockets.

**Figure 4:**
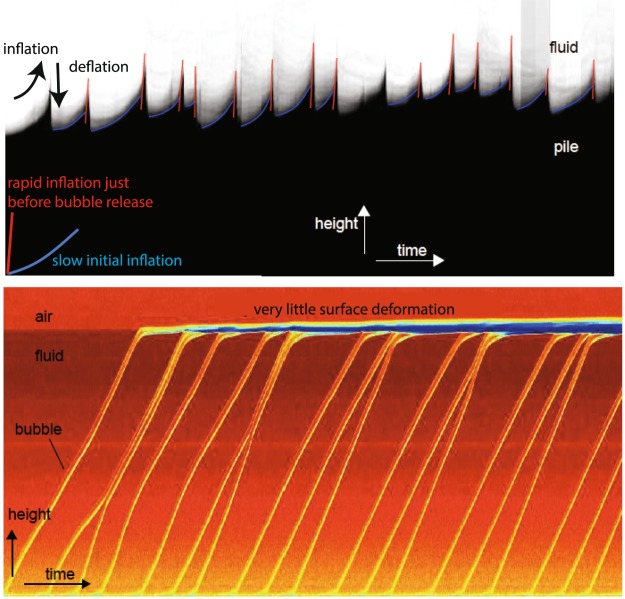
Deformation of the particle pack surface caused by gas pockets rising through it. (**a**) Images of a vertical slice through the experimental tank versus time. Deformation of the top surface of the particle pack occurs when gas pockets rise through the particle pile. Blue lines show slow expansion of the pile while the gas pocket is relatively low in the pile; red lines show rapid acceleration of the inflation just before gas pocket release into the liquid layer above. (**b**) The particle-free experiment shows gas bubbles rising up through a liquid at a near-constant rate, until they reach the air layer at the top of the liquid. Little surface deformation can be seen.

The upper surface of the particle pack exhibits deformation during rise of the gas pockets (Fig. 4a), in contrast to the particle-free, liquid-only case (Fig. 4b). There is slow inflation of the top of the particle pack when the gas pockets are low in the particle pack, which then accelerates to rapid inflation as the gas pockets near the top surface. The pack deflates rapidly once the gas pocket escapes into the liquid layer overlying the particle pack. In contrast, for the particle-free case, bubbles rise at a constant rate through the liquid and escape at the top into an air layer, without causing any deformation of the top of the liquid layer (Fig. 4b).

With a higher gas flux (Fig. 3b), the injected gas has a higher pressure, and forms a more extensive fracture-like channel which rises through the particle pack. The continuing gas supply maintains the pressure within this channel, leading to deformation of the pack around the channel until it breaks through to the surface, the pack relaxes, and a new channel develops. At even higher fluxes (>~1 mL/s), the fracture-like channel becomes more stable, and no longer collapses after reaching the surface. A steady stream of gas flows along this channel, which spans the height of the particle pile and is of order ~1 mm wide. While a channel is stable, the pile ceases to deform periodically, and gradually compacts (Fig. 3b). Since the channel is thinner than the width of the Hele-shaw cell, it cannot be seen except for where it exits the top of the particle pack. The channel may eventually become unstable and collapse, leading to a further phase of periodic gas emission (‘pulsing’ on Fig. 3b) until a new stable channel forms. Sometimes, only the upper part of the channel collapses, leading to localised formation of gas intrusions.

**Figure 5:**
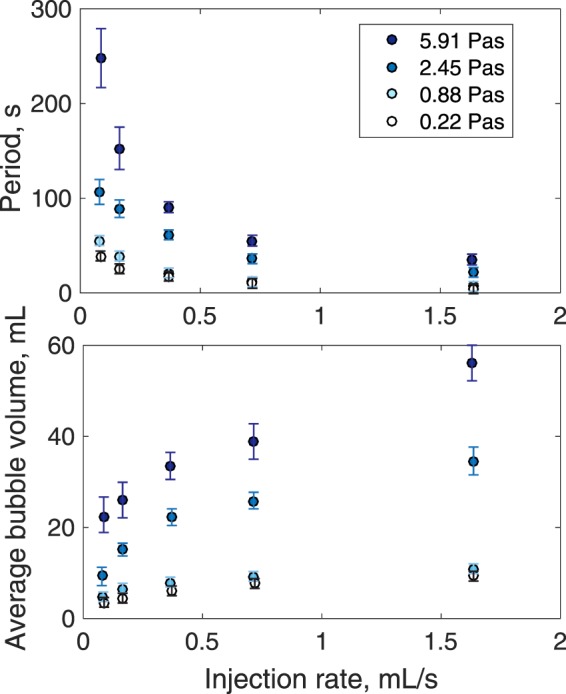
Cyclic release of gas at the top of the particle pack. Variation of the frequency and size of gas emissions with gas flux and viscosity. Frequency and bubble volume both increase with increasing input gas flux and viscosity of interstitial liquid.

Gas release at the top of the particle pack is cyclic, particularly for low injection rates (Fig. 5). The mean period of gas emission episodes and the volume of gas released at the top of the particle pile varies with injection rate and with liquid viscosity. As the injection rate of air into the base of the particle pile increases, the period of the gas release events at the top of the pile decreases (Fig. 5a), which is expected since higher gas flow rates will increase the rate at which the bubble grows to a critical size to rise through the pile, leading to more frequent gas slugs. Figure 5b shows that the volume of gas released during each episode of gas pocket release at the top of the particle pack increases with gas injection rate and with viscosity. Period and mean bubble colume both increase with viscosity, an effect which can be collapsed by normalizing by the lowest viscosity.

In the experiments, the local effective tensile strength of the pack, σ, typically has magnitude of order 50–500 Pa^41,42^. As a gas intrusion grows and deepens, the hydrostatic pressure difference, and hence overpressure on the top surface of the intrusion, also increases. Eventually, with a gas pocket 1–2 cm deep, the hydrostatic pressure jump, Δ*ρgh* ~ 100–200 Pa (where Δ*ρ* is the density difference between the magma and the gas, *g* is the acceleration of gravity and *h* is the vertical extent of the bubble), is sufficient to overcome the effective tensile strength of the pack, and a gas-filled channel advances into the overlying pack. The local deformation of the particle pack produces temporal and spatial variations in the packing of the particles and hence changes the conditions at which successive intrusions form. We note that in the close-packed particle system, surface tension (measured for our natrasol solution to be 66 mN^−1^, see methods) impedes the motion of the gas through the pore spaces between the packed particles, but by opening up a channel through tensile failure of the pack, the effect of the surface tension becomes much smaller and the gas can rise through the system.

### Applications of our results to natural magmatic-volcanic systems

Since the processes we observe relate to the collective macroscopic motion of the particle pack, rather than that of individual particles, we propose that the phenomena explored here may also arise on the larger scale of a magmatic mush system. We propose that the existence of an effective tensile strength of a crystal mush may rationalise observations of the episodic gas release during strombolian explosions. For the example of Stromboli volcano, for example, volcanic gases during the regular explosions at Stromboli are largely composed of carbon dioxide and have been proposed to be sourced from deeper than ~4 km in the conduit system^5,7^. Although gas that is generated deeper in the plumbing system may be supplied to the mush in the form of discrete slugs produced from foam collapse or two-phase flow instabilities^43^, our experiments suggest that a crystalline mush present in the shallow plumbing system at 2–4 km depth^8^ may itself act as a valve, leading to episodic gas release at the surface even with a more continuous deep source. Our experiments support recent numerical modelling^16^ which suggests that periodic eruptions may be driven by failure of a crystal-rich ‘plug’, although we believe that the crystal-rich layer may be substantially deeper than envisaged by^16^, in order to explain the CO_2_-rich nature of the gas slugs^7^. In this new picture, the size of the gas pocket, which later becomes a gas slug in the shallow conduit, depends on the overpressure which can be sustained in a gas intrusion prior to tensile failure and formation of a channel. The overpressure, *∆p*, at the top of a localised gas intrusion within the mush depends on the vertical extent, *h*, of the intrusion and the density contrast of the gas and the particle pack, *∆ρ* so that *∆p* = *∆ρgh*. The effective tensile strength depends on the particle concentration and packing, but is typically in the range of 100–10,000 Pa^41,44,45^. Our mechanism of gas flow through fractures differs from recently proposed models of viscous fingering and capillary flow, with no mush deformation, to explain gas transport through crystal-rich magma^38^ and emerges as a result of the relatively low strength of such particle packs.

The variation of effective tensile strength within the particle pile is a key feature of granular suspensions, and fundamentally distinguishes it from the behaviour of a purely fluid non-Newtonian system^46,47^. As explored by numerical modeling^48^, the resistance to plastic deformation as the particles becomes jammed depends on a number of factors, most importantly the particle packing (described by a coordination number) and the lubrication of inter-particle contacts, largely controlled by liquid viscosity. Jamming can occur at crystal volume fractions lower than the maximum packing fraction if there is a breakdown of lubrication of the particle contacts, which can occur for higher liquid viscosities and shear rates. The intermittent stalling and ascent of the gas pockets at different levels within the particle pile is indicative of this complex behaviour.

We propose that our experiments highlight dependencies of gas behaviour on liquid viscosity not fully explored in previous studies. An increase in liquid viscosity (for a fixed particle fraction) promotes plastic deformation and causes an increase in the size of the gas intrusions (Fig. 5b); jamming of the particles and the transition to fracture seems to happen later than for a lower liquid viscosity. One explanation for this is that, with a higher liquid viscosity, bubbles can grow to larger sizes by plastically deforming the particle pile prior to reaching the effective tensile strength, beyond which the gas can rise by fracturing the overlying particle pile. Conversely, at lower fluid viscosity, particle contacts are less well lubricated and more dominated by friction, so the resistance to plastic deformation is greater and jamming occurs earlier while the bubbles are smaller. Higher injection rates also lead to larger gas intrusions. We propose that at higher injection rates, the shear rates are larger and this has the effect of increasing the lubrication of particle contacts. Like the effect of higher viscosity, this reduces the resistance to plastic deformation and so leads to larger gas intrusions. Our data point to the interesting theoretical challenge of quantifying this changing pattern of deformation in such viscous particle-fluid packs.

At Stromboli Volcano, bubble radii, at pressures of order 75 MPa in the crystal-laden magma have been estimated (from gas emission rates) to be 0.8–1.0 m^49^. With a mush density of ~2680 kgm^−3^, a gas pocket of this size would produce a hydrostatic pressure of ~10^4^ Pa. To form a pocket this size would require a local tensile strength of order ~0.01 MPa, consistent with the measured ranges mentioned above. Our experiments suggest that increases in eruption frequency might occur with an increase in gas production rate (Fig. 5), since the higher gas flow requires larger pressures to drive the deformation. This observation is supported by thermal, seismic and infrasonic data collected at Stromboli, where vigorously degassing periods (with a higher gas flux) are marked by more frequent bubble bursts or weak strombolian explosions, whereas lower gas flux periods are marked by less frequent “puffing” activity^50^.

To conclude and summarize, we propose a novel mechanism for explaining how the cyclicity in strombolian eruptions is derived. We propose that the interaction between an exsolved volatile phase and crystal-laden magma provides the fundamental basis for segregating and accumulating gas pockets in crystal-rich regions of magmatic plumbing systems, thereby allowing gas-rich strombolian eruptions to occur when these gas pockets rise to shallow conduits and form slugs. The magmas from which this gas is derived ultimately become subsumed into cumulate bodies in the edifice or upper crust beneath such volcanoes. The CO_2_-rich gas migrating through the magmatic system itself plays an important role in increasing the crystallinity of overlying magmas thorugh extensive CO_2_-fluxing, which dehydrates magmas and causes extensive crystallisation.

## Methods

A particle pack of depth 40 cm was set up between two backlit, parallel sheets of Perspex, 1 cm apart, and filled with a solution of Natrasol polymer and water, of density 1000 kg/m^3^ (Fig. 1). Four different solutions were used, with viscosities of 0.22, 0.88, 2.45 and 4.59 Pa s. Temperature was kept constant at 18–20 C. Surface tension was measured for the natrosol solutions using drop shape analysis. Both solutions were found to have similar surface tension to water. The particles (plastic spheres of radius 1 mm) have density 1290 kg/m^3^ which leads to a Stokes settling speed of order 10^−5^ m/s in the solution of Natrasol, and the packing fraction is 60–65 vol%. Air was supplied at the base of the particle pile using a calibrated peristaltic pump to supply a known range of gas fluxes (0.097, 0.187, 0.387, 0.717, 1.647, 3.407, 4.927, 7.627 mL/s). High-resolution digital photographs recorded the motion and deformation of the particle pile in response to the gas flux and image analysis was carried out using a customised matlab code.

A challenge of this set of experiments was the persistence of tiny bubbles trapped in the particle pack. These bubbles were introduced during mixing of the natrosol solution and particles and, due to the solution’s high viscosity, were difficult to remove. The solution was to fill the tank with particles first, and then pump the fluid in from beneath. When the fluid was injected slowly enough, it percolated through the pore space like a porous gravity flow, with no deformation of the pile. This process took several hours, so due to time constraints, five experiments were carried out in order of flow rate using the same pile. The pile was left to settle for 20 minutes between successive experiments.
